# Brachial insertion of fully implantable venous catheters for chemotherapy: complications and quality of life assessment in 35 patients

**DOI:** 10.1590/S1679-45082016AO3606

**Published:** 2016

**Authors:** Igor Yoshio Imagawa Fonseca, Mariana Krutman, Kenji Nishinari, Guilherme Yazbek, Marcelo Passos Teivelis, Guilherme André Zottele Bomfim, Rafael Noronha Cavalcante, Nelson Wolosker

**Affiliations:** 1 Fundação Antônio Prudente, Hospital A. C. Camargo, São Paulo, SP, Brazil.; 2 Hospital Israelita Albert Einstein, São Paulo, SP, Brazil.

**Keywords:** Catheters, indwelling/adverse effects, Drug therapy, Patient satisfaction, Patient safety

## Abstract

**Objective:**

To prospectively evaluate the perioperative safety, early complications and satisfaction of patients who underwent the implantation of central catheters peripherally inserted via basilic vein.

**Methods:**

Thirty-five consecutive patients with active oncologic disease requiring chemotherapy were prospectively followed up after undergoing peripheral implantation of indwelling venous catheters, between November 2013 and June 2014. The procedures were performed in the operating room by the same team of three vascular surgeons. The primary endpoints assessed were early postoperative complications, occurring within 30 days after implantation. The evaluation of patient satisfaction was based on a specific questionnaire used in previous studies.

**Results:**

In all cases, ultrasound-guided puncture of the basilic vein was feasible and the procedure successfully completed. Early complications included one case of basilic vein thrombophlebitis and one case of pocket infection that did not require device removal. Out of 35 patients interviewed, 33 (94.3%) would recommend the device to other patients.

**Conclusion:**

Implanting brachial ports is a feasible option, with low intraoperative risk and similar rates of early postoperative complications when compared to the existing data of the conventional technique. The patients studied were satisfied with the device and would recommend the procedure to others.

## RESUMO

**Objetivo:**

Avaliar prospectivamente segurança perioperatória, complicações precoces e grau de satisfação de pacientes submetidos ao implante de cateteres centrais de inserção periférica pela veia basílica.

**Métodos:**

Foram acompanhados prospectivamente e submetidos ao implante de cateteres de longa permanência de inserção periférica, entre novembro de 2013 e junho de 2014, 35 pacientes consecutivos com doença oncológica ativa necessitando de quimioterapia. Os procedimentos foram realizados em centro cirúrgico por uma mesma equipe composta por três cirurgiões vasculares. Os desfechos primários avaliados foram as complicações pós-operatórias precoces, ocorridas em até 30 dias após o implante. A avaliação do grau de satisfação foi realizada com base na aplicação de um questionário específico já utilizado em estudos prévios.

**Resultados:**

Em todos os casos, a punção ecoguiada da veia basílica foi possível, e o procedimento foi concluído com sucesso. As complicações precoces observadas incluíram um caso de tromboflebite de basílica e um de infecção de bolsa, ambos tratados clinicamente sem necessidade de retirada do dispositivo. Dos 35 pacientes interrogados, 33 (94,3%) recomendariam o dispositivo para outras pessoas.

**Conclusão:**

A implantação do *port* braquial é uma opção factível, com baixo risco intraoperatório e taxas semelhantes de complicações pós-operatórias imediatas quando comparada a dados já existentes da técnica convencional. Os pacientes estudados apresentaram-se satisfeitos com o dispositivo e recomendariam o procedimento para outras pessoas.

## INTRODUCTION

Long-term central venous access is essential for patients requiring chemotherapy. The fully implantable indwelling catheter known as port-cath, port-a-cath, or simply port is currently the device of choice for this purpose and presents high success rates of insertion.^1-3^

Fully implantable catheters can be inserted through the superior vena cava system, by catheterization of deep (internal jugular, subclavian, innominate, and axillary) or superficial (external jugular, cephalic, and basilic) veins.^4^ Exceptionally, the femoral or great saphenous veins can also serve as access, when there is thrombosis of the superior vena cava system.^5^

When these devices are inserted into the cervical veins of the superior vena cava system, the reservoir is most often positioned in the anterior chest region. When inserted via lower limb veins, the reservoir can be implanted in the abdomen, anterior to the iliac crest or in the femoral region.^6^

An alternative to such catheters is using a peripherally inserted fully implantable catheter introduced via the basilic vein or other axial vein of the arm^7-12^ with the reservoir positioned in the arm. The potential benefits that justify a more detailed study of this technique include reducing the risk of intraoperative complications such as pneumothorax or hemothorax, noninterference in breast imaging, easier access to puncture, and better cosmetic results.

To the extent of our knowledge, there are no reports or case series in the literature documenting complications and satisfaction and quality of life outcomes related to the implantation of ports inserted via peripheral veins and positioned in the arm.

## OBJECTIVE

To prospectively evaluate perioperative safety, early complications and satisfaction of cancer patients requiring chemotherapy who underwent the implantation of central catheters peripherally inserted via basilic vein.

## METHODS

This study was approved by the Ethics Committee of the *Hospital A. C. Camargo Cancer Center*, under number 1.878/14 and all patients signed the informed consent. A total of 35 consecutive patients with active malignancy requiring chemotherapy were prospectively followed up and submitted to the implantation of peripherally inserted indwelling catheters, between November 2013 and June 2014. The inclusion criteria were age greater than or equal to 18 years; malignancy; and the need for a catheter used exclusively for chemotherapy. The study excluded patients using anticoagulation; with basilic vein with diameter of less than 2mm; with prior history of indwelling catheter implant in the superior vena cava system; and evolving to death in less than 30 days. The measurement of the transverse vein diameter (in its major axis) was performed immediately before the procedure using ultrasound in two-dimensional mode.

### Implantation technique

All procedures were performed in the operating room by the same team of three vascular surgeons experienced in venous access and accompanied by an anesthesiologist. Most patients had favorable clinical conditions and received sedation and local anesthesia (88.67%). In three cases (8.57%), only local anestesia was used, due to unfavorable clinical conditions, and in one case, the patient underwent general anesthesia. Whenever possible, the access route of choice was the non-dominant arm, and the basilic vein was used for catheter implantation. In patients with breast cancer submitted to lymphadenectomy or in cases of basilic vein with less than 2mm in diameter, the limb with more favorable access conditions was chosen, regardless of being the dominant arm.

The preparation for surgery included shaving the catheter implantation site when necessary, and disinfection of the entire limb with 2% chlorhexidine, followed by antisepsis with alcoholic chlorhexidine. We did not use antibiotics on induction or postoperatively.

The polyurethane catheter used in all study cases was the Medcomp^®^ Dignity^®^ CT 6.6F model (Harleysville, Pennsylvania, USA) which has a radiopaque low profile reservoir.

Local anesthesia was performed with 2% xylocaine and 2% ropivacaine. The basilic vein was punctured using echo-guided technique anda 21-gauge needle, followed by the insertion of a 0.018” guidewire. A small incision, was made at the puncture site, and the 6.6F introducer was inserted into the vein guided by fluoroscopy.

After removal of the guidewire and introducer, the catheter was inserted and directed to the central position, also under radioscopic view, and the sheath was peeled away. The catheter was tunneled from the puncture site to a subcutaneous pocket made with an incision approximately 3cm long, located in the distal third of the arm, 3 to 4cm above the elbow crease.

After cutting it to an adequate length, the catheter was connected to the reservoir implanted in the pocket. The reservoir was then fixed to the muscular plane, with 4.0 nylon sutures.

The reservoir incision was closed in two planes with absorbable sutures in the subcutaneous layer, and intradermal skin sutures. The incision on the puncture site was closed using only an intradermal suture.

Intraoperative data, such as operating time, type of anesthesia, access route changes and intraoperative complications (pneumothorax, hemothorax, vascular injury, cardiac arrhythmia, and hematoma) were recorded for further evaluation.

**Figure 1:**
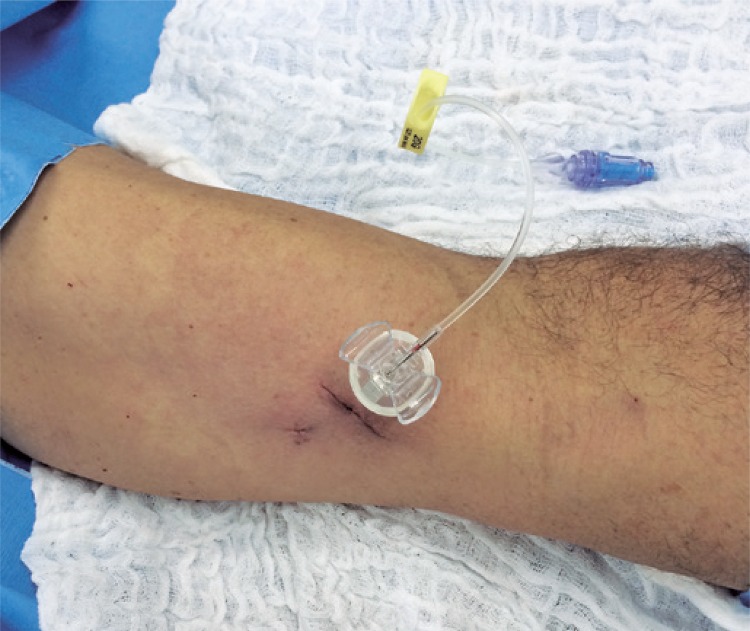
Immediate postoperative result of punctured port

Patients were instructed to keep applying sterile occlusive dressings for 3 days after the procedure. In case of need for immediate use of the device, the puncture was performed in surgical room, as shown in figure 1.

### Follow up

Patients included in the sample were clinically evaluated 10 and 30 days after the procedure, or at any other time of the study in case of any catheter-related intercurrent events.

Additional tests such as X-ray or Doppler ultrasound were requested only if the patient complained of symptoms related to the catheter *(e.g*., dysfunction, edema or changes related to the surgical wound).

The primary outcomes assessed were the early postoperative complications, defined as events occurring within 30 days after implantation.

A satisfaction questionnaire already used and validated in previous publications^8^ was applied in the second evaluation, after 30 days of implantation. From the patients’ perspective, this questionnaire analyzes data involving recognition of the need for the device; aspects of comfort; anxieties generated by the use of the device; interference in daily activities; aesthetics and overall satisfaction based on the recommendation grade indicated by the patient.

The patients were asked whether they agreed or disagreed with statements relating to the different aspects of satisfaction analyzed. The results of the questionnaire were compared to the findings available in the study presented by Goltz et al.^8^ for brachial ports, using the same analysis tool.

### Statistical analysis

Comparisons of our questionnaire results and the findings of Goltz et al.^8^ were performed using χ^2^ frequency test or Fisher’s exact test. When p>0.05, we considered the results (frequencies) of the study consistent with the findings already published. When p<0.05, we considered that our study was in disagreement with the formerly published study.

We had no comparative data in the study presented by Goltz et al.^8^ for three questions in the questionnaire: one related to the indication of the device and two associated with the implantation and with comfort aspects. This occurred because these parameters were not evaluated by the authors 30 days after the procedure, but only immediately after implantation.

## RESULTS

A total of 35 catheters were implanted in 35 patients. In all cases, the basilic vein puncture was possible, and the procedure was successfully completed. Table 1 shows the patients’ demographic data and the type of cancer being treated.

**Table 1:** Demographic data and the type of cancer

Number of implanted catheters	n=35
Sex	
Female	18
Male	17
Age	
Mean	62
Median	59.0
Type of cancer	
Gastrointestinal tract	20
Head and neck	5
Lung	3
Breast	1
Gynecological (not breast)	2
Hematological	2
Sarcomas (retroperitoneal/limbs)	2

The mean and median times for the procedure were respectively 36 and 35 minutes. A total of 31 ports were implanted in the left basilic vein, and only 4 in the right basilic vein. In only one case, a contrast injection (15mL) was necessary, because the guidewire was not advancing to the central position due to tortuosity in the superior vena cava system.

No significant intraoperative complications were observed. There was only one case of local ecchymosis in the subcutaneous pocket region, which did not cause major complications.

Regarding the early postoperative complications (up to 30 days after implantation), we observed a case of basilic vein thrombophlebitis associated with the catheter, which was clinically treated with anticoagulants, not requiring removal of the device.

In one case we observed the formation of a seroma around the reservoir in the subcutaneous pocket, with the leakage of a small amount of serous fluid through the puncture needle hole. For this patient, a pressure dressing was applied for 1 week, with satisfactory resolution.

By the end of follow-up, we observed a case of pocket infection, treated with ciprofloxacin for 7 days, and it was not necessary to remove the device.

**Figure 2:**
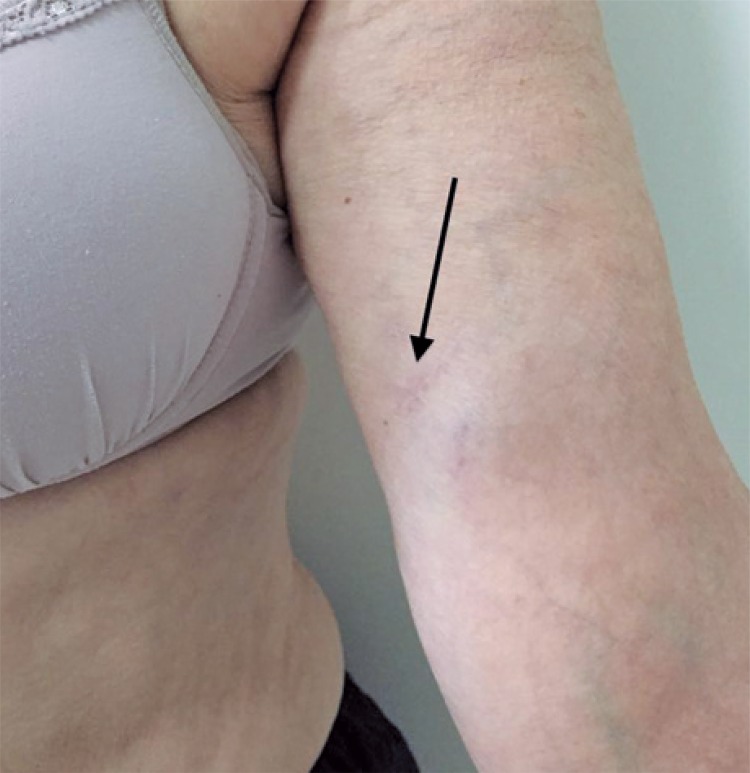
Final esthetic result three months after the procedure (arrow shows the surgical scar site)

Table 2 shows the satisfaction questionnaire results with the brachial port for the 35 patients in the study compared with the findings of Goltz et al.^8^Of the 35 patients interviewed, 33 (94.3%; p=0.22) would recommend the device to others. In almost all cases (97.1%), the patients were satisfied with the type of anesthesia used and reported no discomfort during the implantation procedure. In more than 88.6% of cases, the catheter with brachial implantation site did not interfere significantly in the daily activities. The aesthetic result was considered positive in 88.6% of patients, which is discordant with the study presented by Goltz et at.^8^ (p=0.002). Figure 2 shows the final aesthetic result three months after the procedure.

**Table 2:** Results of the questionnaire on satisfaction level (patient’s perspective) compared to the study by Goltz et al.(8)

	Study		Study by Goltz et al.^(8)^ 30 days (brachial port)		p value
	
	I agree n (%)	I disagree n (%)	I agree n (%)	I disagree n (%)	
Indication and device					
I know why the catheter was implanted	32/35 (91.4)	3/35 (8.6)	-	-	
I know how the device works	24/35 (68.6)	11/35 (31.4)	25/25(100.0)	0/25 (0.0)	0.002
Implant and comfort aspects					
Discomfort during the procedure was acceptable	34/35 (97.1)	1/35 (2.9)	-	-	
I would prefer general to local anesthesia if I were submitted to the procedure again	2/35 (5.7)	33/35 (94.3)	-	-	
I would prefer repeated peripheral venous punctures to having a port device	7/35 (20.0)	28/35 (80.0)	0/25 (0.0)	25/25(100.0)	0.035*
The port causes an unpleasant feeling	7/35 (20.0)	28/35 (80.0)	15/25 (60.0)	10/25 (40.0)	0.002
The port causes pain	5/35 (14.3)	30/35 (85.7)	6/25 (24.0)	19/25 (76.0)	0.338
After a certain period, I did not feel the port any longer	25/35 (71.4)	10/35 (28.6)	15/25 (60.0)	10/25 (40.0)	0.355
The injection of large volumes fast is important for me, for instance for contrast-enhanced tests	10/35 (28.6)	25/35 (71.4)	17/25 (68.0)	8/25 (32.0)	0.002
The access to the catheter is painful	15/35 (42.9)	20/35 (57.1)	6/25 (24.0)	19/25 (76.0)	0.131
Anxiety feelings (“I fear the port...”)					
Will not work one day	13/35 (37.1)	22/35 (62.9)	13/25 (52.0)	12/25 (48.0)	0.252
Will be displaced	9/35 (25.7)	26/35 (74.3)	4/25 (16.0)	21/25 (84.0)	0.368
Can be damaged while I play with children	10/35 (28.6)	25/35 (71.4)	6/25 (24.0)	19/25 (76.0)	0.693
Can be damaged when someone hugs me	8/35 (22.9)	27/35 (77.1)	5/25 (20.0)	20/25 (80.0)	0.791
Can be a source of infection	13/35 (37.1)	22/35 (62.9)	10/25 (40.0)	15/25 (60.0)	0.822
Impact on daily life activities (“The catheter disturbs me if...”)					
I have a shower	4/35 (11.4)	31/35 (88.6)	4/25 (16.0)	21/25 (84.0)	0.608
I have a bath in the tub	3/35 (8.6)	32/35 (91.4)	4/25 (16.0)	21/25 (84.0)	0.377
I exercise	3/35 (8.6)	32/35 (91.4)	7/25 (28.0)	18/25 (72.0)	0.046
I move my arms	3/35 (8.6)	32/35 (91.4)	10/25 (40.0)	15/25 (60.0)	0.004
I put on clothes or take them off	3/35 (8.6)	32/35 (91.4)	10/25 (40.0)	15/25 (60.0)	0.004
I drive my car (wearing a seat belt)	2/35 (5.7)	33/35 (94.3)	1/25 (4.0)	24/25 (96.0)	0.764
I wear bra	0/35 (0)	18/35 (51.4)	0/12 (0.0)	12/12(100.0)	-
I try to seat on a certain position so that the catheter cannot move	5/35 (14.3)	30/35 (85.7)	1/25 (4.0)	24/25 (96.0)	0.190
Cosmetic aspects					
The scar worries me	4/35 (11.4)	31/35 (88.6)	12/25 (48.0)	13/25 (52.0)	0.002
General satisfaction					
I would recommend other people to implant a catheter like mine	33/35 (94.3)	2/35 (5.7)	25/25(100.0)	0/25 (0.0)	0.224

p value obtained by the χ^2^ frequencies test. * p value obtained by the Fisher’s exact test.

## DISCUSSION

Indwelling catheters are devices of great importance widely used for the administration of chemotherapy to cancer patients worldwide.^9^ They provide comfort, convenience and security in the application of chemotherapy, which when administered via peripheral vein, may present complications, such as phlebitis, pain and even more severe consequences, like skin necrosis and limb compartment syndrome due to extravasation of medication. These complications ultimately delay the proposed treatment and cause unnecessary concern, affecting the quality of life of cancer patients.

The sites most commonly used for the insertion of these devices are currently the veins of the superior vena cava system (internal jugular and subclavian) with the reservoir positioned in the anterior chest region. These techniques are proven safe^10^ and have become even more effective after the systematic use of two-dimensional ultrasound, with a significant reduction in cannulation failure rates, inadvertent puncture of carotid, and hematoma formation, when compared to the technique based on the use of anatomical landmarks.^11^ The incidence of complications, such as hemothorax or pneumothorax, is also drastically reduced after the echo-guided puncture technique is mastered, a goal usually achieved after 5/10 cases/surgeon.^12^

Brachial insertion ports are safely implanted in peripheral veins, especially the basilic vein, with easy maintenance and low morbidity, since the rates of severe perioperative complications related to puncture or pneumothorax and hemothorax are zero. Risks associated with catheter fracture between the clavicle and the first rib (pinch-off syndrome) that occur with subclavian vein implantation also appear to be reduced by the use of this technique_._^9^

Devices with the reservoir implanted in the arm offer an interesting alternative for patients with gross tumors or exposure to radiation therapy in cervical and/or anterior chest regions which contraindicate the port implantation in the conventional position, avoiding femoral vein catheterization, greatly associated with infectious complications.^13^ Another possible advantage of the brachial port insertion includes better cosmetic results, avoiding scars in more exposed and visible regions.

We observed one (2.9%) case of thrombotic complication and one (2.9%) case of infectious complication in the cases studied. Recent studies show that the rates of thrombotic and infectious complications related to ports implanted in the anterior chest region range from zero to 3.2%^3,9,12,14-16^ and from 1.1 to 9%,^3,9,12,15-17^ respectively. Our initial study sample, although small, showed data consistent with the literature, suggesting equivalence of techniques.

The hypothesis of higher risk of thrombotic complications in brachial ports due to the small size of the basilic vein and the greater length of the catheter would be a potential disadvantage of this technique when compared to cervical insertion devices. Whereas initial studies disclosed venous thrombosis rates ranging between zero and 26%,^17,18^ the current literature shows more favorable results which are consistent with our findings, demonstrating thrombosis rates ranging between zero and 4.5%.^9,19^

The only thrombotic complication was a superficial thrombophlebitis of the basilic vein in a female patient, confirmed by Doppler ultrasound performed in the first week after device implantation, due to complaint of limb edema. This patient was 51 years old and had rectal cancer. After one week of full anticoagulation, she had complete remission of the edema, and the treatment was continued for 3 months with no complications. The catheter remained functioning throughout the treatment period.

Due to the small size of the basilic vein when compared to the veins of the superior vena cava system, the thinnest needle puncture is crucial to ensure success in the puncture for peripheral access. The use of a 21-gauge needle prevents hematoma formation and vein spasms that complicates new punctures and even render them unfeasible, when catheterization is not achieved on the first attempt.

We found significant differences for some questions addressed in the questionnaire compared to the findings of Goltz et al.^8^ Our patients had a lower level of knowledge about the functioning of the device, and 20% reported preference for repeated peripheral venopunctures to insert the port - an assertion that was not supported by any patient of the comparative study. Moreover, in our study the patients complained less about any unpleasant sensation associated with the port, and reported lower impact caused by the device on daily activities, such as moving the arm and wearing clothes. The level of satisfaction with the aesthetic results observed among patients in our study was higher (88.6% *versus* 52.0%; p=0.002).

Our patients did not consider important the rapid injection function of large volumes through the catheter, as for contrast-enhanced imaging exams with injection pump. This can be explained by the fact that there is no contrast infusion routine via port in radiological studies conducted in our service.

In current literature, there are larger series reporting the outcomes of arm implanted ports. The largest study involved 154 patients.^20^ No studies have simultaneously assessed the complications and quality of life outcomes, from the patient’s perspective. Our results showed a high level of patient satisfaction on quality of life with the brachial catheter insertion, and almost all the patients analyzed would recommend this device to others.

Our study demonstrated only an initial evaluation of a technique not often used in our practice, but that can be employed safely and presents satisfactory results. It can be used as an alternative in situations that preclude the reservoir implantation in the normal position, or provided as a first choice, depending on the patient’s preference. There is a lack of comparative studies on the implantation of catheters in the arm *versus* the veins of the superior vena cava system, and these studies are currently being studied by our group.

## CONCLUSION

The brachial port implantation is a feasible option with low risk surgery and similar rates of immediate postoperative complications compared to existing data of the conventional technique. The patients studied were satisfied with the device and would recommend the procedure to others.
